# Mapping of Gene Expression Reveals *CYP27A1* as a Susceptibility Gene for Sporadic ALS

**DOI:** 10.1371/journal.pone.0035333

**Published:** 2012-04-11

**Authors:** Frank P. Diekstra, Christiaan G. J. Saris, Wouter van Rheenen, Lude Franke, Ritsert C. Jansen, Michael A. van Es, Paul W. J. van Vught, Hylke M. Blauw, Ewout J. N. Groen, Steve Horvath, Karol Estrada, Fernando Rivadeneira, Albert Hofman, Andre G. Uitterlinden, Wim Robberecht, Peter M. Andersen, Judith Melki, Vincent Meininger, Orla Hardiman, John E. Landers, Robert H. Brown, Aleksey Shatunov, Christopher E. Shaw, P. Nigel Leigh, Ammar Al-Chalabi, Roel A. Ophoff, Leonard H. van den Berg, Jan H. Veldink

**Affiliations:** 1 Department of Neurology, Rudolf Magnus Institute of Neuroscience, University Medical Center Utrecht, Utrecht, The Netherlands; 2 Department of Genetics, University Medical Center Groningen, Groningen, The Netherlands; 3 Groningen Bioinformatics Centre, Groningen Biomolecular Sciences and Biotechnology Institute, University of Groningen, Haren, The Netherlands; 4 Department of Human Genetics, David Geffen School of Medicine, University of California Los Angeles, Los Angeles, California, United States of America; 5 Department of Biostatistics, David Geffen School of Medicine, University of California Los Angeles, Los Angeles, California, United States of America; 6 Department of Epidemiology and Biostatistics, Erasmus University Medical Center Rotterdam, Rotterdam, The Netherlands; 7 Department of Neurology, University Hospital Leuven, University of Leuven, Leuven, Belgium; 8 Laboratory for Neurobiology, Vesalius Research Centre, Flanders Institute for Biotechnology (VIB), Leuven, Belgium; 9 Department of Clinical Neuroscience, Umeå University, Umeå, Sweden; 10 Department of Neuropediatrics, University of Paris, Bicetre Hospital, Paris, France; 11 Department of Neurology, Université Pierre et Marie Curie, Hôpital de la Salpêtrière, Paris, France; 12 Department of Neurology, Beaumont Hospital, Dublin, Ireland; 13 Department of Neurology, Trinity College, Dublin, Ireland; 14 Department of Neurology, University of Massachusetts School of Medicine, Worcester, Massachusetts, United States of America; 15 Department of Neurology, Massachusetts General Hospital, Charlestown, Massachusetts, United States of America; 16 Medical Research Council Centre for Neurodegeneration Research, King’s College London, Department of Clinical Neuroscience, Institute of Psychiatry, London, United Kingdom; 17 Department of Medical Genetics, Rudolf Magnus Institute of Neuroscience, University Medical Center Utrecht, Utrecht, The Netherlands; 18 Center for Neurobehavioral Genetics, University of California Los Angeles, Los Angeles, California, United States of America; Genentech, United States of America

## Abstract

Amyotrophic lateral sclerosis (ALS) is a progressive, neurodegenerative disease characterized by loss of upper and lower motor neurons. ALS is considered to be a complex trait and genome-wide association studies (GWAS) have implicated a few susceptibility loci. However, many more causal loci remain to be discovered. Since it has been shown that genetic variants associated with complex traits are more likely to be eQTLs than frequency-matched variants from GWAS platforms, we conducted a two-stage genome-wide screening for eQTLs associated with ALS. In addition, we applied an eQTL analysis to finemap association loci. Expression profiles using peripheral blood of 323 sporadic ALS patients and 413 controls were mapped to genome-wide genotyping data. Subsequently, data from a two-stage GWAS (3,568 patients and 10,163 controls) were used to prioritize eQTLs identified in the first stage (162 ALS, 207 controls). These prioritized eQTLs were carried forward to the second sample with both gene-expression and genotyping data (161 ALS, 206 controls). Replicated eQTL SNPs were then tested for association in the second-stage GWAS data to find SNPs associated with disease, that survived correction for multiple testing. We thus identified twelve *cis* eQTLs with nominally significant associations in the second-stage GWAS data. Eight SNP-transcript pairs of highest significance (lowest p = 1.27×10^−51^) withstood multiple-testing correction in the second stage and modulated *CYP27A1* gene expression. Additionally, we show that *C9orf72* appears to be the only gene in the 9p21.2 locus that is regulated in *cis*, showing the potential of this approach in identifying causative genes in association loci in ALS. This study has identified candidate genes for sporadic ALS, most notably *CYP27A1*. Mutations in *CYP27A1* are causal to cerebrotendinous xanthomatosis which can present as a clinical mimic of ALS with progressive upper motor neuron loss, making it a plausible susceptibility gene for ALS.

## Introduction

Amyotrophic lateral sclerosis (ALS) is a neurodegenerative disease characterized by progressive muscle weakness caused by loss of central and peripheral motor neurons. Symptoms typically have a localized limb or bulbar onset and progress to other muscle groups of the body. Denervation of respiratory muscles and dysphagia leading to respiratory complications are the most common causes of death. There is no cure for this rapidly progressive disease.

Approximately 5% of patients have a family history of ALS [1]. All other cases are considered to have a sporadic form of the disease. ALS is considered to be a disease of complex etiology with both genetic and environmental factors contributing to disease susceptibility [2]. These genetic factors are the subject of extensive research [3]. Multiple genome-wide association studies (GWAS) and candidate gene studies have been carried out, implicating several genes in the susceptibility to ALS [4]–[8], but attempts to replicate most of these genes have proven difficult [9]–[13]. Recently, our group has published a GWAS comprising over 4,800 patients and nearly 15,000 controls and identifying *UNC13A* and 9p21.2 as susceptibility loci for sporadic ALS [7]. The 9p21.2 locus was recently replicated in an independent set of British patients and controls [12] and also shown to be strongly associated with ALS in Finland [14]. This locus was previously found to be one of the linked loci in families with ALS and frontotemporal dementia (FTD), and it was recently shown that a hexanucleotide repeat expansion in *C9orf72* was the basis of this linkage signal [15], [16].

Despite these large study samples, GWAS have been able to explain only little of the genetic variation in ALS [4]–[7]. An important drawback of GWAS is the burden of multiple-testing correction, requiring even larger sample sizes in order to be able to detect small effects. It is common practice to apply a strict Bonferroni correction to GWAS data. With so many tests, there is a high false-negative rate, as true associations are hidden in the fog of random associations.

It has been established that gene expression levels can be mapped to genomic variation as a quantitative trait in order to detect so-called expression quantitative trait loci (eQTLs) [17]–[19]. Recently, it has been shown that trait-associated SNPs are more likely to be eQTLs [20], making the systematic analysis of eQTLs in the context of a GWAS a promising tool for the discovery of novel disease-causing genes. In addition, eQTLs can have local and distant effects, allowing for the identification of parts of biological networks related to disease. These networks might be the link between several different genetic variants that appear to be associated with a disease in a GWAS [19]. In practical terms, in order to identify eQTLs associated with disease, both genome-wide genotype data as well as genome-wide gene expression levels have to be collected. The focused genetic mapping of gene expression levels has frequently been applied to the fine-mapping of risk loci resulting from GWAS, for example in the study of asthma [21] and Crohn’s disease [22]. Furthermore, genome-wide eQTL analysis has proven fruitful in the study of diseases including obesity [23], hypercholesterolemia [24], celiac disease [25], and late-onset Alzheimer disease [26]. In the present study, we have performed a genome-wide screen for eQTLs associated with susceptibility to ALS.

**Figure 1:**
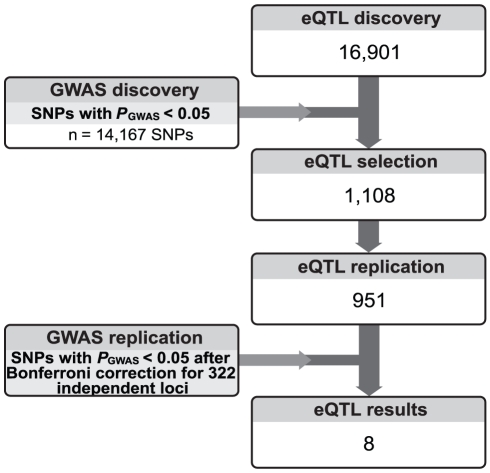
Study design. For each step, the number of resulting SNP-transcript pairs in *cis* is shown.

A schematic overview of our study design is shown in Figure 1. We performed an initial screen for eQTLs in an eQTL discovery set. The eQTL SNPs resulting from this screen that had a nominally significant effect in a discovery set from our previously published GWAS [7] were selected for follow-up in the eQTL replication set. Ultimately, replicated eQTLs were tested for significant effects in the GWAS replication data, correcting for multiple testing.

## Methods

### Ethics Statement

All participants gave written informed consent and approval was obtained from the Institutional Review Board of the University Medical Center Utrecht. The present study was conducted according to the principles expressed in the Declaration of Helsinki.

### GWAS Data

Genome-wide genotype data were derived from a previously published GWAS of sporadic ALS in seven countries (The Netherlands, Belgium, France, Ireland, United Kingdom, Sweden, United States) [7]. All patients fulfilled the 1994 El Escorial criteria for probable or definite ALS [27]. Cohorts for which genome-wide SNP data were available were included. For both the discovery and replication set, genotype files with Illumina Beadchip data (HumanHap 300K, HumanCNV 370K, HumanHap 550K or HumanHap 610K platforms) were merged and the following quality control measures were taken. Only SNPs common to all cohorts were used. Triallelic and C/G or A/T SNPs were excluded. Genotype files were merged, and after each merge, a *flipscan* (scan for possible allele swaps) was performed in PLINK v1.07 [28]. SNPs with call rate <95%, minor allele frequency <5%, deviation from Hardy-Weinberg equilibrium in controls (p<1×10^−4^), or with differing heterozygosity or missing rates between cases and controls were excluded. Duplicate samples, samples with a genotyping rate <95%, samples without gender information, or samples where the genotypic gender did not match the phenotype file gender were excluded. LD-based SNP pruning was used to determine a subset of SNPs in approximate linkage equilibrium. This subset of SNPs was used to identify related samples, which were subsequently removed (pi-hat >0.2). The software package EIGENSTRAT was used to detect population substructure by principal components analysis [29]. HapMap phase III release 2 genotypes were added into this analysis in order to determine population outliers. After removal of population outliers, new principal components were calculated. More detailed data on included subjects, genotyping methods, and quality control are available in Text S1 and Table S5.

### Expression Data

Genome-wide gene expression data were obtained from 805 Dutch individuals (357 patients and 448 controls), who were also genotyped on either the HumanHap 300K, HumanCNV 370K or HumanHap 550K platforms in the previously described GWAS [7]. Patients were recruited at our referral clinic for motor neuron disease at the University Medical Center Utrecht, The Netherlands. Included patients were diagnosed with probable or definite sporadic ALS according to the 1994 El Escorial criteria [27]. Messenger RNA was collected and extracted from peripheral whole blood using PAXgene tubes and PAXgene extraction kit (Qiagen). Samples were hybridized to Illumina HumanHT-12v3 Expression BeadChips. Case and control samples were randomly assigned to the chips and all chips were run in one batch. Before quality control, expression levels were available for 48,803 probes. Raw expression data were quantile normalized and log_2_ transformed [30] in R (2009, The R Foundation for Statistical Computing). Using principal components analysis of expression data, outlier arrays were detected. Non-pseudoautosomal Y chromosome transcript expression levels were used for a gender check. Outlier arrays, samples with inconsistent gender information, and samples designated as duplicates in our GWAS data, were removed from the raw data (n = 67). Also, non-autosomal probes were excluded (n = 2,002). The thus obtained trimmed raw dataset was again quantile normalized and log_2_ transformed. All probe sequences were aligned to the NCBI build 36 reference genome using UCSC’s Genome Browser function BLAT [31]. Non-specific probes, defined as no or multiple hits with a sequence homology >95%, were removed (n = 7,234). RefSeq (updated on 27 September 2010) and UniGene (build #228, release date 29 October 2010) databases were used to determine probes mapping to transcripts designated as *retired* and these probes were excluded as well (n = 2,449), leaving 37,118 gene-expression probes.

### eQTL Datasets

For the genetic mapping of gene expression, the subset of Dutch individuals with both genome-wide genotype and expression data was tested for population substructure by principal components analysis of genomic data using EIGENSTRAT [29]. By inspecting the first two principal components, two outlier samples (one case, one control) were identified and excluded. Subsequently, new principal components were calculated. Non-autosomal SNPs were removed from the eQTL analysis. We randomly split our expression dataset to form equally sized discovery and replication sets (Table S1).

### Statistical Analysis

For the GWAS data, association with disease was tested in a logistic model using gender, dummy-coded nationality and the first eight principal components in order to correct for ancestry as covariates. To determine the number of principal components to be included in the logistic regression model, the first ten principal components from the EIGENSTRAT [29] analysis were tested for association with case/control status (threshold p<0.05). For the GWAS discovery set, eight principal components were included in the logistic model, while for the GWAS replication set two principal components were included. Analyses were performed in PLINK v1.07 [28] and R (2009, The R Foundation for Statistical Computing).

For all analyses involving expression data, Surrogate Variable Analysis (SVA) was used to account for heterogeneity in gene expression due to known and unknown environmental, technical or demographic factors [32]. SVA captures these factors into covariates for use in statistical models. Additionally, ‘riluzole use’ status was obtained, the only drug available to ALS patients with proven effect on survival.

For the eQTL analyses, SNP genotypes coded as an additive genetic model were tested for association with gene expression by linear regression using disease status, age, gender, surrogate variables (18 in the discovery set and 19 in the replication) and riluzole use as covariates. *Cis* eQTLs were defined as SNPs modulating transcript expression levels within a region of 1Mb surrounding a probe’s genomic midpoint [26]. False-positive *cis* effects may, however, occur due to SNPs that are located within a transcript probe or that are in linkage disequilibrium (LD) with SNPs mapping within a transcript probe [33]. We used the Broad Institute SNAP tool v2.2 [34] to determine pairwise LD between *cis* effect SNPs and SNPs mapping to a transcript probe in either of the HapMap phase III release 2 or 1000 Genomes Pilot 1 CEU panels. 21,863 SNP-transcript combinations (pairwise LD threshold *r^2^* >0.2) were excluded from analysis. Similarly, we removed 24,170 SNP-transcript combinations with an InDel overlapping with a transcript probe, according to the Database of Genomic Variants (version 10, November 2010) [35]. There were 3,541,781 possible SNP-transcript combinations in *cis* left for analysis. The number of possible combinations in *cis* was used for Benjamini-Hochberg false discovery rate (FDR) calculations. Significant *cis* effects were those SNP-transcript pairs that had significant p values at an FDR of 5% after 10,000 permutations. Permutations were performed swapping case/controls labels so that each subject is assigned the genotype vector of another random subject, while the expression matrix is unchanged. This prevents the underestimation of the null distribution, thereby preventing the detection of false-positive eQTLs, as described previously [36]. Analyses were performed in PLINK [28] and R (2009, The R Foundation for Statistical Computing).

### eQTL Selection

In order to link the identified eQTLs to disease, we made a selection of significant *cis* effects in the eQTL discovery set. Recent studies on the genetics of gene expression have shown that disease-associated loci from GWAS are greatly enriched for eQTLs [20], [25]. Thus, we selected SNP-transcript pairs that had a nominal SNP p value <0.05 in our GWAS discovery data (Figure 1).

Only these SNP-transcript pairs were used for follow-up in the replication data. Patient characteristics for the expression replication dataset are presented in Table S1. SNP genotypes were correlated to gene expression levels following a similar statistical analysis as used for our discovery set. Again, a 5% FDR significance threshold was applied.

Subsequently, association with ALS for SNPs from the replicated *cis* SNP-transcript pairs was tested in the GWAS replication data by logistic regression using gender, dummy-coded nationality and the first two EIGENSTRAT principal components (these were significantly correlated to case/control status) as covariates. Association test results were clumped based on LD (*r^2^* >0.5) using PLINK, so that SNP p values could be obtained for independent eQTLs. eQTLs with a replication p_GWAS_ <0.05 after Bonferroni correction for the number of independent (LD-based clumped) loci were considered to be significant (Figure 1).

## Results

### eQTL Discovery

After quality control, eQTL analyses were performed on 162 ALS cases and 207 controls in the eQTL discovery set with data on 261,682 autosomal SNPs and 37,118 expression probes. Patient characteristics are summarized in Table S1. At a Benjamini and Hochberg false discovery rate (FDR) of 5%, we detected 16,901 significant SNP-transcript pairs in *cis* (Figure 1).

### GWAS Discovery

In the GWAS discovery set, 2,261 ALS cases and 8,328 patients remained after quality control measures with genotypes for 268,952 SNPs. Details of included study populations are shown in Table S2. Association analysis resulted in one SNP (rs12608932 in gene *UNC13A*) with genome-wide significance (p = 1.7×10^–8^) after Bonferroni correction for 268,952 SNPs. A Manhattan plot of genome-wide results is shown in Figure S1. A quantile-quantile plot of disease association p values is provided in Figure S2 (genomic control λ = 1.03). There were 14,167 autosomal SNPs with a nominal p value <0.05. These SNPs were used to prioritize eQTLs found in the eQTL discovery set (Figure 1).

From the eQTL discovery results, we selected the 1,108 SNP-transcript pairs (755 eQTL SNPs) in *cis* with discovery p_GWAS_ <0.05 (Figure 1). To confirm the hypothesis that disease-associated SNPs are more likely to be *cis* eQTLs [20], we searched for enrichment for eQTLs in our list of SNPs with p_GWAS_ <0.05. We first determined the number of *cis* eQTLs in the set of SNPs with p_GWAS_ <0.05 (n = 755). Then, we randomly selected a subset of 14,167 SNPs with p_GWAS_ >0.05, matched for minor allele frequency to the set of SNPs with p_GWAS_ <0.05 (in 5% frequency bins). Subsequently, we determined the number of eQTLs present in each of these sets of SNPs, using 100,000 permutations. By determining how often more than the initial number of eQTLs were observed, we showed that there was evidence for enrichment for eQTLs in the set of disease-associated SNPs (empirical p = 0.003).

### eQTL Replication

The eQTL replication set comprised 161 ALS patients and 206 control samples (Table S1). 951 out of 1,108 selected SNP-transcript pairs in *cis* were significantly replicated (Figure 1). The eQTL SNPs of these SNP-transcript pairs were selected for replication in the GWAS replication data.

### GWAS Replication

After quality control, there were 1,307 ALS cases and 1,835 controls in the GWAS replication set with genotypes for 266,492 SNPs (Table S2). 577 *cis* eQTL SNPs were tested for association in the GWAS replication data. Using linkage disequilibrium-based clumping of association results [28], 322 independent clumps could be formed. This number of clumps was used for Bonferroni correction, as these clumps designate independent loci. Table 1 shows clumps with a nominal p_GWAS_ <0.05 in the replication set. Ultimately, we identified 1 *cis* eQTL, comprising 8 SNP-transcript pairs, which was significantly replicated, and the transcript of which mapped to gene *CYP27A1*. The results for this locus are listed in Table S3, also indicating that the explained variance of gene expression that is achieved by the linear models ranged from 48–65%. The relationships between the SNPs and gene-expression levels are shown in Figure S3.

**Table 1:** eQTLs with a nominally significant GWAS p value in the replication data.

Locus	Chr	Illumina HT-12v3 probe identifier	Clump index SNP	Minor allele	GWAS discovery SNP association		GWAS replication SNP association			Joint GWAS SNP association		eQTL p value after permutations		eQTL direction of effect
					OR	p	OR	p	p bonf.	OR	p	Discovery	Replication	
*CYP27A1*	2	ILMN_1704985	rs4674345	G	1.08	0.049	1.23	1.32×10^−4^	0.042	1.12	1.84×10^−4^	1.65×10^−46^	1.19×10^−47^	+
*CENPV*	17	ILMN_1729142	rs10491104	G	1.11	3.79×10^−3^	1.17	3.64×10^−3^	n.s.	1.14	2.35×10^−5^	1.15×10^−5^	9.50×10^−4^	+
*SLC11A1*	2	ILMN_1741165, ILMN_1735737	rs2279014	A	1.12	2.26×10^−3^	1.15	0.011	n.s.	1.13	4.98×10^−5^	5.48×10^−27^	7.49×10^−40^	+
*TTC39C*	18	ILMN_1746720	rs1154227	G	1.08	0.037	1.15	0.011	n.s.	1.12	3.00×10^−4^	6.23×10^−6^	5.25×10^−4^	+
*SPI1, MYBPC3*	11	ILMN_1696463, ILMN_1781184	rs7126210	A	1.08	0.044	1.15	0.021	n.s.	1.11	2.28×10^−3^	5.44×10^−9^	1.57×10^−5^	+
*RABEP1*	17	ILMN_1719622	rs3865351	A	0.91	0.024	0.88	0.021	n.s.	0.90	2.06×10^−3^	2.70×10^−7^	1.19×10^−6^	+
*ZNF586*	19	ILMN_2372200	rs4801516	A	0.92	0.020	0.89	0.027	n.s.	0.92	8.15×10^−3^	6.73×10^−5^	3.60×10^−3^	+
*KIAA0513*	16	ILMN_1693233	rs8056742	G	1.17	7.51×10^−3^	1.19	0.029	n.s.	1.19	1.42×10^−4^	4.29×10^−8^	6.44×10^−15^	+
*C17orf75, CDK5R1*	17	ILMN_1797155, ILMN_1730928	rs4795700	A	1.12	2.15×10^−3^	1.12	0.034	n.s.	1.11	4.14×10^−4^	3.33×10^−26^	9.85×10^−40^	+
*SLC39A1*	1	ILMN_2116714	rs11264743	A	0.92	0.032	0.88	0.035	n.s.	0.91	2.72×10^−3^	9.47×10^−7^	2.09×10^−4^	+
*Hs.447737*	5	ILMN_1896967	rs13354021	G	0.92	0.040	0.89	0.040	n.s.	0.91	3.46×10^−3^	3.05×10^−7^	1.51×10^−4^	+
*CLEC12A*	12	ILMN_1663142, ILMN_2292178	rs10505745	A	1.16	1.91×10^−3^	1.14	0.049	n.s.	1.14	5.75×10^−4^	5.09×10^−5^	6.81×10^−5^	–

Independent eQTLs are based on LD-based SNP clumping. For each locus, the clump index SNP (with the lowest p value) is shown. For the GWAS replication results, Bonferroni corrected p values are given for the testing of 322 clumps. SNP association results in the joint GWAS data were based on a total of 3,568 ALS patients and 10,163 controls. For the eQTL direction of effect, ‘+’ means the SNP minor allele was associated with increased expression levels, ‘–’ means decreased gene expression. Chr, chromosome; LD, linkage disequilibrium; GWAS, genome-wide association study; OR, odds ratio; p bonf., Bonferroni corrected p value; n.s., not significant; eQTL, expression quantitative trait locus.

### Fine-mapping of Loci *UNC13A* and Chromosome 9p21.2

In addition to our genome-wide screen for eQTLs associated with sporadic ALS, we specifically examined possible relevant *cis* effects in two previously associated loci (gene *UNC13A* and chromosome 9p21.2) [7], [12]. The detection of *cis* effects might fine-map these loci. For the *UNC13A* locus (SNP rs12608932), multiple-testing correction was applied for 41 possible SNP-transcript pairs in *cis* (as determined by a genomic distance of <500kb between the SNP and a probe’s midpoint). One SNP-transcript pair had a nominal p value <0.05, the transcript of which mapped to gene *PGLS* (p_EQTL_ = 0.01). However, when using a 5% Benjamini-Hochberg FDR for the locus as multiple-testing correction, no SNP-transcript pairs reached statistical significance. For the chromosome 9p21.2 locus, we looked for *cis* eQTLs within a 130kb LD block comprising previously associated SNPs (rs2814707 and rs3849942). Multiple-testing correction for the testing of 328 SNP-transcript pairs was applied using a 5% FDR. Two SNP-transcript pairs reached the threshold for statistical significance and were associated with *C9orf72 isoform a* expression levels (Table 2 and Figure S4). SNP rs1565948 modulated *C9orf72* gene expression in both eQTL discovery and replication sets and was associated with susceptibility to ALS in the joint GWAS data; however, no association with ALS was found in the GWAS replication set alone (Table 2).

**Table 2:** Results for fine-mapping of loci previously associated with ALS.

Locus	Illumina HT-12v3 probe identifier	SNP	Minor allele	LD with rs3849942	GWAS discovery SNP association		GWAS replication SNP association		Joint GWAS SNP association		eQTL p value after permutations		Expression variance explained (R^2^)
				*r^2^, D'*	OR	p	OR	p	OR	p	Discovery	Replication	Combined data
*C9orf72 isoform a*,Chr. 9	ILMN_1741881	rs10122902	A	0.08, 1.00	0.97	0.49	0.98	0.81	0.97	0.42	1.39×10^−7^	2.08×10^−4^	0.80
		rs1565948	G	0.32, 0.99	1.14	3.17×10^−4^	1.01	0.93	1.11	6.00×10^−4^	5.00×10^−5^	3.00×10^−4^	0.80

The minor allele of rs10122902 was associated with increased *C9orf72* expression levels, while the minor allele of rs1565948 was associated with decreased expression. LD estimates with SNP rs3849942 and SNP association results in the joint GWAS data were based on a total of 3,568 ALS patients and 10,163 controls. The expression explained variance (R^2^) was estimated from expression data from both discovery and replication eQTL datasets combined. *C9orf72*, chromosome 9 open reading frame 72; Chr., chromosome; LD, linkage disequilibrium; GWAS, genome-wide association study; OR, odds ratio; eQTL, expression quantitative trait locus.

## Discussion

**Figure 2:**
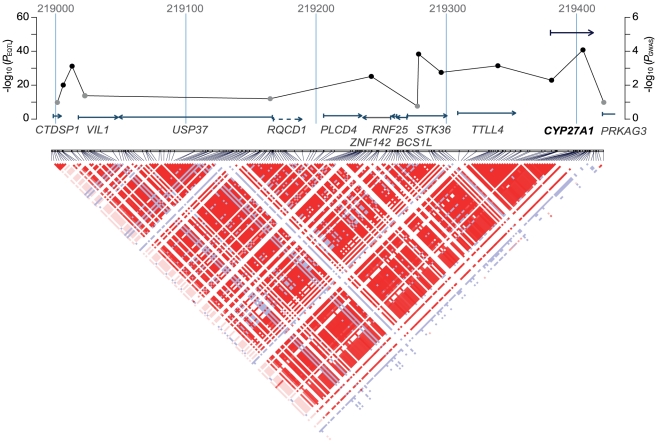
Regional linkage disequilibrium (LD) near the *CYP27A1* locus on chromosome 2. Top: the position of GWAS SNPs and RefSeq genes located within the regional LD block are drawn. On the *X*-axis, genomic position in kb, aligned to NCBI genome build 36 coordinates. On the left *Y*-axis, −log_10_(p values) for the strongest *cis* eQTL association for a gene in the replication data, the vertical position of genes (drawn as arrows) are aligned to this axis and thus represent statistical significance. For one gene (*RQCD1*), no SNP-transcript pair and, therefore, no eQTL p value was available in our data. This gene is shown as a dashed arrow. On the right *Y*-axis, −log_10_(p values) from the replication GWAS analysis for SNPs within the region (black line), SNPs modulating *CYP27A1* expression are shown as black dots, other SNPs are grey. Bottom: pairwise linkage disequilibrium for HapMap phase III release 2 SNPs (CEU+TSI populations). The LD plot was created in Haploview v4.2 [50], using the standard *D'*/LOD color scheme.

The present study reports the results of a large and comprehensive genome-wide screening of the genetics of gene expression in an attempt to find novel genetic variants that associate with sporadic ALS. We used a two-stage approach to minimize the chance of false-positive findings, both for eQTL discovery purposes and for the detection of novel SNP-ALS associations. eQTLs were used for prioritizing GWAS results, as it has been established that SNPs that are truly associated with disease are more likely to be eQTLs [20], [25], [37]. In the present study, we show that the number of eQTLs is greater than expected by chance (p = 0.003) among the SNPs with a nominal association with ALS, compared to frequency-matched SNPs, also indicating that eQTLs may be useful in the prioritization of GWAS results in ALS. We identified eight SNPs in one *cis* eQTL, modulating *CYP27A1* gene expression levels, which replicated in the second eQTL dataset and second GWAS set. The eQTL SNPs within this locus are part of a large linkage disequilibrium (LD) block comprising a total of ten genes (Figure 2). The figure clearly shows that the strongest eQTL associations exist for SNPs modulating *CYP27A1* expression, explaining up to 65% of variation in gene expression of this gene. Additionally, we show that *C9orf72* appears to be the only gene in the 9p21.2 locus that is regulated in *cis,* showing the potential of this approach in identifying causative genes in association loci in ALS.

As shown in Table S3, the SNPs modulating transcript levels had small effect sizes in our joint GWAS association results, the highest odds ratio (OR) being 1.13. We used PS v3.0 [38] for statistical power calculations to determine the required sample size for a third genotypic replication of such SNPs. In order to replicate an association for one SNP with minor allele frequency 0.35 at α = 0.05, one would require a minimum of 2,250 cases and 2,250 controls to achieve 80% power for detecting an effect with OR 1.13. As shown in Table 1, several eQTL SNPs did not reach Bonferroni corrected significance in the replication data alone, but do show stronger effects in the joint GWAS data, indicating that statistical power of the GWAS replication set might be a limiting factor. By testing these SNPs in a third independent replication cohort, additional true associations may be detected. The required sample size for such an effort would, however, increase dramatically when adding more tests. Further international collaboration, therefore, is needed in order to achieve sufficient statistical power for the replication of SNPs with small effect sizes.

We searched MEDLINE, Gene Ontology and OMIM databases to identify links to known pathways in ALS pathogenesis for *CYP27A1*. The *CYP27A1* gene is involved in cholesterol metabolism and has been associated with cerebrotendinous xanthomatosis (CTX), which can present with progressive upper motor neuron signs and is a known clinical mimic for primary lateral sclerosis [39], [40]. Two heterozygous mutations in *CYP27A1* have been reported in a patient with atypical CTX and frontotemporal dementia characteristics [41]. Furthermore, previously, serum cholesterol levels have been implicated in modifying survival and in the onset of respiratory impairment in ALS patients [42]–[44]. The combination of our results and these prior data make *CYP27A1* a plausible candidate gene for ALS.

The strengths of our study are the meticulous pruning of expression probes as present on the expression array, with regard to non-specific mapping in the human transcriptome, or harboring SNPs that might interfere with hybridization of probes to the array, resulting in false-positive eQTLs [33]. In addition, permutation schemes were applied, preserving the LD structure within subjects, also minimizing the detection of false-positive eQTLs. Finally, a two-stage approach, both for eQTLs discovery purposes and for the detection of novel SNP-ALS associations, ensures robustness of the results.

A drawback of the present study lies in the use of whole blood instead of neuronal tissue for the measurement of mRNA expression levels. As neuronal tissue is inaccessible in living ALS patients, one could consider the use of human neuronal tissue from autopsy. However, in post-mortem material of ALS patients, most affected motor neurons will have degenerated and one would be investigating exclusively end-stage disease expression profiles. We have investigated the proportion of overlapping eQTLs between our study and other studies, including two studies on human brain tissue (Table S4) [24], [26], [45], [46]. Studies of the genetics of gene expression appear to have modest overlap in the eQTLs identified. For example, 36.1% of genes mapped by a *cis* eQTL in lymphocytes were identified in a study using lymphoblastoid cell lines [24], [45]. A smaller overlap (22%) was found between two studies on brain tissue, which may partly be due to low statistical power [26], [46]. In the present study, 37 – 52% of the genes mapped by *cis* eQTLs in human brain tissue studies appeared to be present in our data (Table S4). The proportion of overlap with studies on blood-derived tissues was comparable (41 – 45%). Considering the relatively high concordance of genes mapped by *cis* eQTLs in our screen with those found in human brain tissue, we consider blood to be a valid starting point for genetic mapping of gene expression in ALS. A large collection of central nervous system tissue control samples may, however, further boost the discovery of novel genetic variants that are associated with ALS.

The focused analysis of variants in the chromosome 9p21.2 locus, which was previously associated with ALS [7], [12], did not identify rs2814707 or rs3849942 as eQTL SNPs. We did, however, find evidence of two other SNPs (rs10122902 and rs1565948), located within a large LD block surrounding the previously associated markers, to be correlated with altered expression levels of *C9orf72 isoform a*. SNP rs1565948 was associated with ALS in our joint GWAS data. The rs10122902 variant was not associated with ALS in our joint GWAS, but was previously shown to be part of a haplotype with rs3849942, in which the major allele of rs10122902 was associated with increased risk of ALS [12]. Genetic variation in the chromosome 9p21.2 locus, therefore, appears to be associated with altered gene expression of *C9orf72*. The recent discovery of the intronic hexanucleotide repeat expansion in *C9orf72* on a common haplotype in 9p21.2 linked families with ALS and FTD [15], [16], [47] thus illustrates the potential of the combined use of gene expression and genotyping in search for causative genes in human diseases. The mechanism though of the recently discovered repeat expansion in *C9orf72* remains to be established. There could be a direct effect of expression levels of isoforms of *C9orf72*, or a “trans”-like effect through RNA-toxicity, as shown in other repeat expansions diseases including fragile X-associated tremor/ataxia syndrome (FXTAS) [48]. Other types of experiments are needed to elucidate this mechanism.

In summary, our genome-wide study of the genetics of gene expression has identified one *cis* eQTL for sporadic ALS, which modulates *CYP27A1* expression and additionally points to *C9orf72* in the chromosome 9p21.2 locus as the gene involved in ALS pathogenesis. To further identify eQTLs relevant to ALS, the concomitant analysis of epigenetic and other level -omic data, e.g. proteomic or metabonomic can be used, as recently shown in a model organism [49]. These studies are preferably performed in ‘ALS target tissues’, including post-mortem central nervous system tissues and induced pluripotent stem cells differentiated to a neuronal or glial lineage. Such studies may provide us with more insight into novel pathogenic pathways and networks causal to this devastating disease.

## Supporting Information

Text S1 **GWAS quality control.**
(DOC)

Figure S1 **Manhattan plot of autosomal SNP association p values in the GWAS discovery set.**
(PDF)

Figure S2 **Quantile-quantile plot of observed −log_10_ (p values) versus the expectation under the null for the genome-wide association results in the GWAS discovery set.**
(PDF)

Figure S3 **Plots for SNP genotype vs. expression level correlations for eQTL SNPs modulating** ***CYP27A1*** **expression levels.**
(PDF)

Figure S4 **Plots for SNP genotype vs. expression level correlations for eQTL SNPs modulating** ***C9orf72*** **expression levels.**
(PDF)

Table S1 **Expression study populations.**
(PDF)

Table S2 **GWAS populations and genotyping platforms.**
(PDF)

Table S3 **Results for replicated eQTLs associated with** ***CYP27A1*** **expression levels.**
(PDF)

Table S4 ***cis*** **eQTL overlap with previous studies.**
(PDF)

Table S5 **Details of quality control of genome-wide genotype data.**
(PDF)
