# Supplementary material for: Mapping of Gene Expression Reveals CYP27A1 as a Susceptibility Gene for Sporadic ALS
Source: PLoS One. 2012 Apr 11;7(4):e35333. doi: 10.1371/journal.pone.0035333 (PMC3324559; doi:10.1371/journal.pone.0035333)
Supplement: Text S1 — GWAS quality control. (DOC) [file pone.0035333.s001.doc]

**Text S1. GWAS quality control.**

The following quality control measures were applied to the genome-wide genotype data. Quality control was performed for the discovery and replication data separately.

1. **Merging datasets**Only SNPs common to all datasets were extracted. Tri-allelic SNPs or SNPs with A/T or C/G alleles were removed to prevent the occurrence of allele swaps. Subsequently, datasets were merged per country. After each merge, a *flipscan* was performed using PLINK software to check for possible allele swaps .
2. **Removal of duplicate samples**SNPs on chromosome 22 were used for an identity-by-descent (IBD) analysis in PLINK. Pairs of individuals with a relatedness measure (pi-hat) value >0.9 were considered to be indicative of a duplicate sample. From these pairs, one of the individuals was randomly removed from the data.
3. **SNP marker quality control**SNPs with a minor allele frequency (MAF) <5%, or with a genotyping call rate <95%, or not in Hardy-Weinberg equilibrium in controls (test p<1×10^−4^) were removed.
4. **Sample quality control**Samples where gender was not defined in the phenotype file, or with a genotyping call rate <95% were removed. Additionally, inbreeding coefficients (F) were calculated in PLINK, and samples with high (F>0.05) or low (F<−0.025) heterozygosity rates were excluded.
5. **Differential missingness**SNPs were tested for differing missing data rates between cases and controls, and SNPs with a test p<1×10^−3^ were removed. Subsequently, a haplotype-based test for non-random missing genotype data was performed in PLINK, and SNPs with estimated haplotype frequencies >2%, and a test p<1×10^−10^ were excluded.
6. **Gender check**Genetic gender (based on heterozygosity rates of X chromosome SNPs) was compared to the gender reported in the phenotype file, and samples with mismatches were removed.
7. **Check for relatedness between individuals**

For this analysis, a subset of SNPs in approximate linkage equilibrium was selected by linkage disequilibrium (LD)-based SNP pruning in PLINK. Autosomal SNPs with a genotyping call rate >0.999, MAF >5%, and a 100% call rate per sample were LD-based pruned using PLINK’s default settings. In the discovery data, this resulted in a pruned set of 31,362 SNPs, and in the replication data, this subset consisted of 34,400 markers. The pruned set of markers was used for an IBD analysis in PLINK. For pairs of individuals with a pi-hat value >0.2 the individual with the lowest genotyping rate was removed.

1. **Indentify population substructure**Population substructure was assessed by principal components analysis using the EIGENSTRAT program from the EIGENSOFT v3.0 software package. Genotypes for the previously generated subset of markers were merged with genotypes for different populations included in HapMap phase III release 2 (1,184 individuals), which were used as a reference.
   Plots were generated of the first two principal components, and exclusion thresholds for population outliers were defined by visual inspection. Samples identified as population outliers were removed, and principal components analysis was reapplied to the remaining individuals.

Details of quality control statistics for the GWAS cohorts are summarized in Table S5.

**References**

1. Purcell S, Neale B, Todd-Brown K, Thomas L, Ferreira MAR, et al. (2007) PLINK: a tool set for whole-genome association and population-based linkage analyses. Am J Hum Genet 81: 559-575.

2. Price AL, Patterson NJ, Plenge RM, Weinblatt ME, Shadick NA, et al. (2006) Principal components analysis corrects for stratification in genome-wide association studies. Nat Genet 38: 904-909.
