# Supplementary material for: Mapping of Gene Expression Reveals CYP27A1 as a Susceptibility Gene for Sporadic ALS
Source: PLoS One. 2012 Apr 11;7(4):e35333. doi: 10.1371/journal.pone.0035333 (PMC3324559; doi:10.1371/journal.pone.0035333)
Supplement: Figure S1 — Manhattan plot of autosomal SNP association p values in the GWAS discovery set. (PDF) [file pone.0035333.s002.pdf]

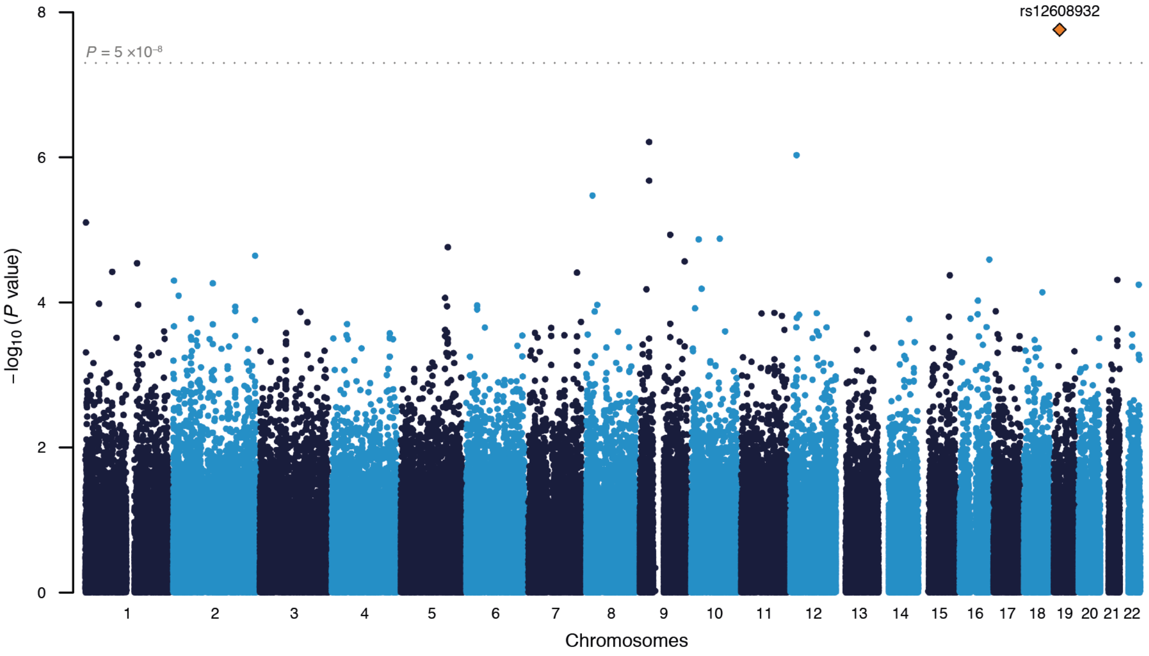

On the *X*-axis genomic positions of SNPs aligned to NCBI genome build 36, chromosome borders are designated by changing dot colors. On the *Y*-axis $-\log_{10}$ (p values) for association between SNP genotype and disease status as obtained from logistic regression analyses in the GWAS discovery set. The dotted line indicates the threshold for genome-wide significance ($p=5\times10^{-8}$). GWAS, genome-wide association study.
