# Supplementary material for: Mapping of Gene Expression Reveals CYP27A1 as a Susceptibility Gene for Sporadic ALS
Source: PLoS One. 2012 Apr 11;7(4):e35333. doi: 10.1371/journal.pone.0035333 (PMC3324559; doi:10.1371/journal.pone.0035333)
Supplement: Figure S2 — Quantile-quantile plot of observed −log10 (p values) versus the expectation under the null for the genome-wide association results in the GWAS discovery set. (PDF) [file pone.0035333.s003.pdf]

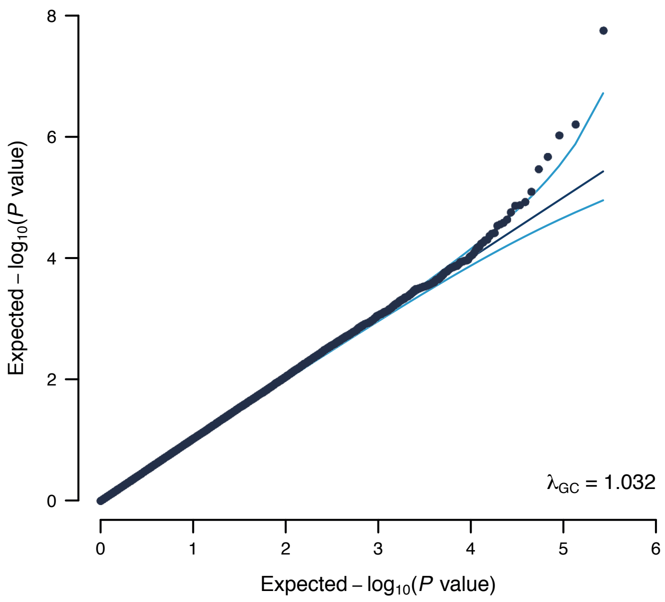

The figure shows departure from the null distribution with λ_{GC}=1.032. GWAS, genome-wide association study.
