# Supplementary material for: Mapping of Gene Expression Reveals CYP27A1 as a Susceptibility Gene for Sporadic ALS
Source: PLoS One. 2012 Apr 11;7(4):e35333. doi: 10.1371/journal.pone.0035333 (PMC3324559; doi:10.1371/journal.pone.0035333)
Supplement: Figure S3 — Plots for SNP genotype vs. expression level correlations for eQTL SNPs modulating CYP27A1 expression levels. (PDF) [file pone.0035333.s004.pdf]

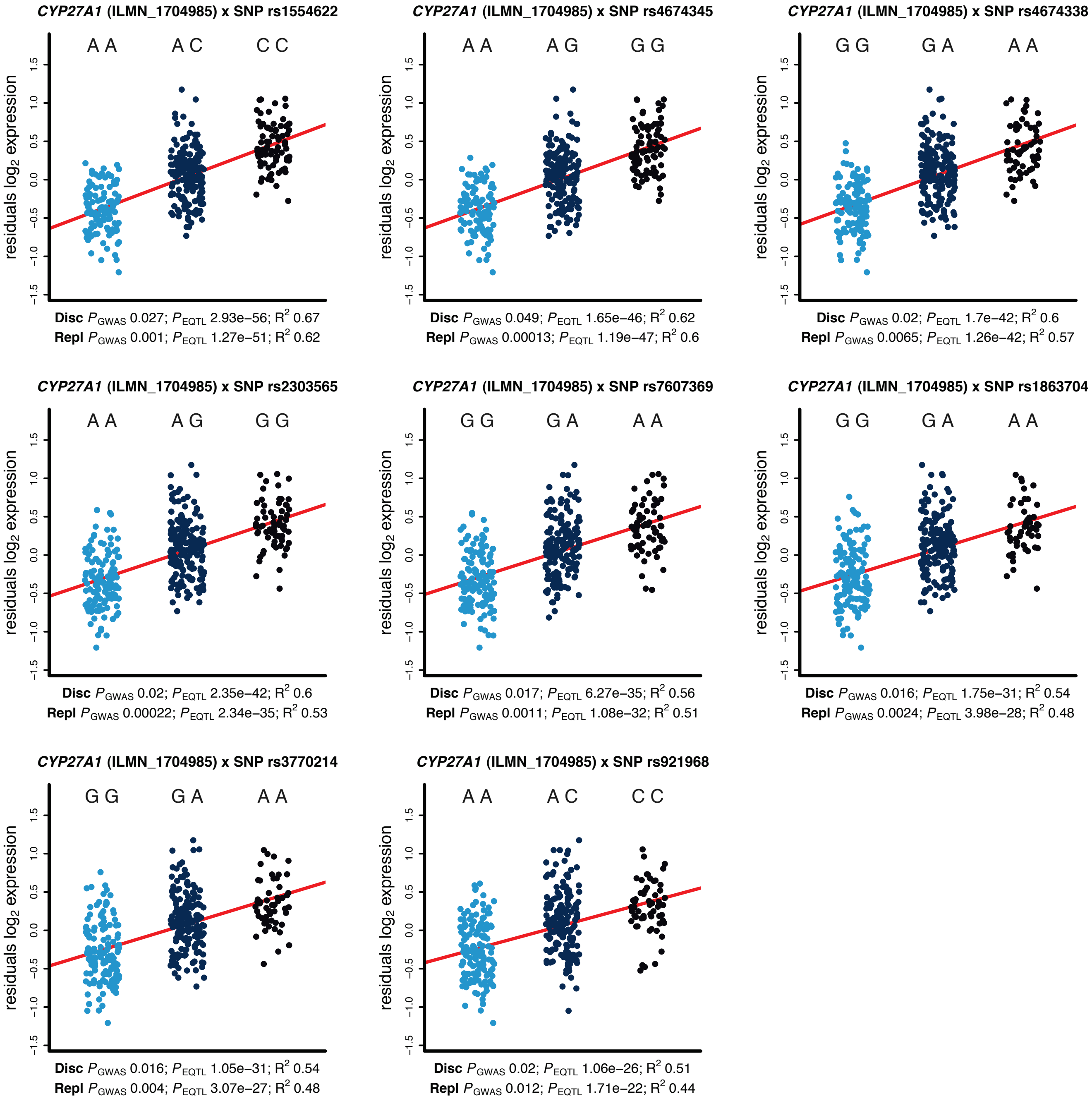

On the *Y*-axis, the residuals of $\log_2$ transformed expression levels for probe ILMN_1704985 mapping to *CYP27A1* after regression of covariates in the replication data. On the *X*-axis SNP genotype bins, according to an additive model; on the left homozygotes for the major allele and homozygotes for the minor allele on the right. A regression line is plotted for each linear model. P values and R^2 (variance explained) for GWAS and eQTL associations in both discovery and replication cohorts are shown below each plot. Disc, Discovery; Repl, Replication; eQTL, expression quantitative trait locus; GWAS, genome-wide association study.
