# Supplementary material for: Mapping of Gene Expression Reveals CYP27A1 as a Susceptibility Gene for Sporadic ALS
Source: PLoS One. 2012 Apr 11;7(4):e35333. doi: 10.1371/journal.pone.0035333 (PMC3324559; doi:10.1371/journal.pone.0035333)
Supplement: Table S1 — Expression study populations. (PDF) [file pone.0035333.s006.pdf]

		Gender, female		Age, mean
	**n**	**n**	**%**	**y**
Discovery				
ALS cases	162	61	38	63.9
Controls	207	90	43	62.7
Replication				
ALS cases	161	67	42	63.6
Controls	206	97	47	62.0
Total	736	315	43	63.2

ALS, amyotrophic lateral sclerosis.
