# Supplementary material for: Mapping of Gene Expression Reveals CYP27A1 as a Susceptibility Gene for Sporadic ALS
Source: PLoS One. 2012 Apr 11;7(4):e35333. doi: 10.1371/journal.pone.0035333 (PMC3324559; doi:10.1371/journal.pone.0035333)
Supplement: Table S2 — GWAS populations and genotyping platforms. (PDF) [file pone.0035333.s007.pdf]

	n ALS cases	n Controls	Platform
Discovery			
The Netherlands	1016	7069	Illumina 317K, 370K, 550K
Belgium	300	328	Illumina 370K
Sweden	458	455	Illumina 370K
Ireland	220	209	Illumina 550K
United States	267	267	Illumina 550K
Total	2261	8328	
Replication			
France	231	709	Illumina 317K
United Kingdom	239	212	Illumina 317K
United States	736	791	Illumina 317K
Ireland	101	123	Illumina 610K
Total	1307	1835	
Joint GWAS	3568	10163	

ALS, amyotrophic lateral sclerosis; GWAS, genome-wide association study.
