# Supplementary material for: Mapping of Gene Expression Reveals CYP27A1 as a Susceptibility Gene for Sporadic ALS
Source: PLoS One. 2012 Apr 11;7(4):e35333. doi: 10.1371/journal.pone.0035333 (PMC3324559; doi:10.1371/journal.pone.0035333)
Supplement: Table S3 — Results for replicated eQTLs associated with CYP27A1 expression levels. (PDF) [file pone.0035333.s008.pdf]

Locus	Illumina probe identifier	SNP	Minor allele	LD with index SNP (rs4674345)	GWAS discovery SNP association		GWAS replication SNP association		Joint GWAS SNP association		eQTL p value after permutations		Expression variance explained (R^2)
				r^2	OR	p	OR	p	OR	p	Discovery	Replication	Combined data
CYP27A1, Chr. 2	ILMN_1704985	rs4674345	G	1	1.08	0.049	1.23	1.32×10^{-4}	1.12	1.84×10^{-4}	1.65×10^{-46}	1.19×10^{-47}	0.62
		rs2303565	G	0.66	1.09	0.020	1.22	2.25×10^{-4}	1.13	8.79×10^{-5}	2.35×10^{-42}	2.34×10^{-35}	0.57
		rs1554622	C	0.79	1.09	0.027	1.19	1.00×10^{-3}	1.11	3.98×10^{-4}	2.93×10^{-56}	1.27×10^{-51}	0.65
		rs7607369	A	0.58	1.09	0.017	1.19	1.06×10^{-3}	1.12	2.61×10^{-4}	6.27×10^{-35}	1.08×10^{-32}	0.55
		rs1863704	A	0.55	1.10	0.016	1.18	2.37×10^{-3}	1.12	2.26×10^{-4}	1.75×10^{-31}	3.98×10^{-28}	0.52
		rs3770214	A	0.54	1.10	0.016	1.17	3.95×10^{-3}	1.12	3.14×10^{-4}	1.05×10^{-31}	3.07×10^{-27}	0.52
		rs4674338	A	0.66	1.09	0.020	1.16	6.46×10^{-3}	1.11	7.51×10^{-4}	1.70×10^{-42}	1.26×10^{-42}	0.59
		rs921968	C	0.39	1.09	0.020	1.15	0.012	1.11	7.88×10^{-4}	1.06×10^{-26}	1.71×10^{-22}	0.48

The direction of effect for all SNP-transcript pairs was the same; for each SNP, the minor allele was associated with increased *CYP27A1* expression levels. LD estimates with SNP rs4674345 and SNP association results in the joint GWAS data were based on a total of 3,568 ALS patients and 10,163 controls. The gene expression explained variance (R^2) was estimated from expression data from both discovery and replication eQTL datasets combined. *CYP27A1*, cytochrome P450, family 27, subfamily A, polypeptide 1; Chr., chromosome; LD, linkage disequilibrium; GWAS, genome-wide association study; OR, odds ratio; eQTL, expression quantitative trait locus; ALS, amyotrophic lateral sclerosis.
