# Supplementary material for: Mapping of Gene Expression Reveals CYP27A1 as a Susceptibility Gene for Sporadic ALS
Source: PLoS One. 2012 Apr 11;7(4):e35333. doi: 10.1371/journal.pone.0035333 (PMC3324559; doi:10.1371/journal.pone.0035333)
Supplement: Table S4 — cis eQTL overlap with previous studies. (PDF) [file pone.0035333.s009.pdf]

Study	Sample size	Tissue	n genes mapped by *cis* eQTLs		Overlapping results	
	n		sign. threshold	n	n	%
This study, two stages	369 + 367	Whole blood	permuted p FDR 0.05	2,211	–	–
Göring *et al.* [1]	1,240	Lymphocytes	LOD score >3	737	332	45.0
Stranger *et al.* [2]	270	Lymphoblastoid cell lines	permuted p<0.001	299	122	40.8
Webster *et al.* [3]	364	Brain cortex	permuted p<0.05	280	103	36.8
Gibbs *et al.* [4]	600	Four regions in human brain	FDR 0.05	281	146	52.0

Comparison of the number of genes mapped by *cis* eQTLs in the present study and four other studies that have looked into the genetics of gene expression. The number of genes mapped by *cis* eQTLs was based on the significance threshold that was used in each of the studies. eQTL, expression quantitative trait locus; FDR, false discovery rate; LOD, logarithm of odds.

References

1. Göring HHH, Curran JE, Johnson MP, Dyer TD, Charlesworth J, et al. (2007) Discovery of expression QTLs using large-scale transcriptional profiling in human lymphocytes. Nat Genet 39: 1208-1216.
2. Stranger BE, Forrest MS, Clark AG, Minichiello MJ, Deutsch S, et al. (2005) Genome-wide associations of gene expression variation in humans. PLoS Genet 1: e78.
3. Webster JA, Gibbs JR, Clarke J, Ray M, Zhang W, et al. (2009) Genetic control of human brain transcript expression in Alzheimer disease. Am J Hum Genet 84: 445-458.
4. Gibbs JR, van der Brug MP, Hernandez DG, Traynor BJ, Nalls MA, et al. (2010) Abundant quantitative trait loci exist for DNA methylation and gene expression in human brain. PLoS Genet 6: e1000952.
