# Supplementary material for: Mapping of Gene Expression Reveals CYP27A1 as a Susceptibility Gene for Sporadic ALS
Source: PLoS One. 2012 Apr 11;7(4):e35333. doi: 10.1371/journal.pone.0035333 (PMC3324559; doi:10.1371/journal.pone.0035333)
Supplement: Table S5 — Details of quality control of genome-wide genotype data. (PDF) [file pone.0035333.s010.pdf]

Dataset		Pre QC			QC SNPs						QC samples						After QC		
**Cohort, country**	**Source**	**ALS**	**CON**	**SNPs**	**Common**	**MAF**	**HWE**	**Call rate**	**Missing ALS/CON**	**Missing by haplotype**	**Dupli-cates**	**Call rate**	**Hetero-zygosity**	**Sex check**	**Related**	**Population outliers**	**ALS**	**CON**	**SNPs**
Discovery																			
Netherlands	UMC Utrecht	461	450	317503	311395	8131	3440	4091	22040	5891	0	3	10	3	3	6	450	436	268952
Netherlands	UMC Utrecht	582	629	370404	311395	8131	3440	4091	22040	5891	8	8	2	5	8	17	566	597	268952
Netherlands	UMC Utrecht	0	5974	561466	311395	8131	3440	4091	22040	5891	14	0	7	3	534	20	0	5396	268952
Netherlands	RS-I cohort, The Rotterdam Study	0	704	561466	311395	8131	3440	4091	22040	5891	1	2	11	32	10	8	0	640	268952
Belgium	University Hospital Gasthuisberg	300	328	370404	311395	8131	3440	4091	22040	5891	3	27	11	8	3	2	300	328	268952
Sweden	Umeå University Hospital	458	455	370404	311395	8131	3440	4091	22040	5891	8	0	23	15	12	22	458	455	268952
Ireland	Beaumont Hospital, Dublin	220	209	561466	311395	8131	3440	4091	22040	5891	0	0	2	0	0	1	220	209	268952
USA	NIH	267	267	555351	311395	8131	3440	4091	22040	5891	3	1	6	0	0	3	267	267	268952
Total		2261	8328		311395	8131	3440	4091	22040	5891	37	41	72	66	570	79	2261	8328	268952
Replication																			
France	Evry	251	724	307790	301686	7735	2060	12579	9839	4571	0	19	6	4	0	6	231	709	266492
UK	King's College London	245	221	307790	301686	7735	2060	12579	9839	4571	0	0	4	6	0	5	239	212	266492
USA	MGH & Atlanta	753	811	307790	301686	7735	2060	12579	9839	4571	0	0	12	11	0	14	736	791	266492
Ireland	Beaumont Hospital, Dublin	103	127	620901	301686	7735	2060	12579	9839	4571	5	0	0	1	0	0	101	123	266492
Total		1352	1883		301686	7735	2060	12579	9839	4571	5	19	22	22	0	25	1307	1835	266492

QC, quality control; ALS, amyotrophic lateral sclerosis; CON, control; MAF, minor allele frequency; HWE, Hardy-Weinberg Equilibrium.
